# Supplementary figures and images for: PhyloPrimer: a taxon-specific oligonucleotide design platform
Source: PeerJ. 2021 Apr 29;9:e11120. doi: 10.7717/peerj.11120 (PMC8098674; doi:10.7717/peerj.11120)

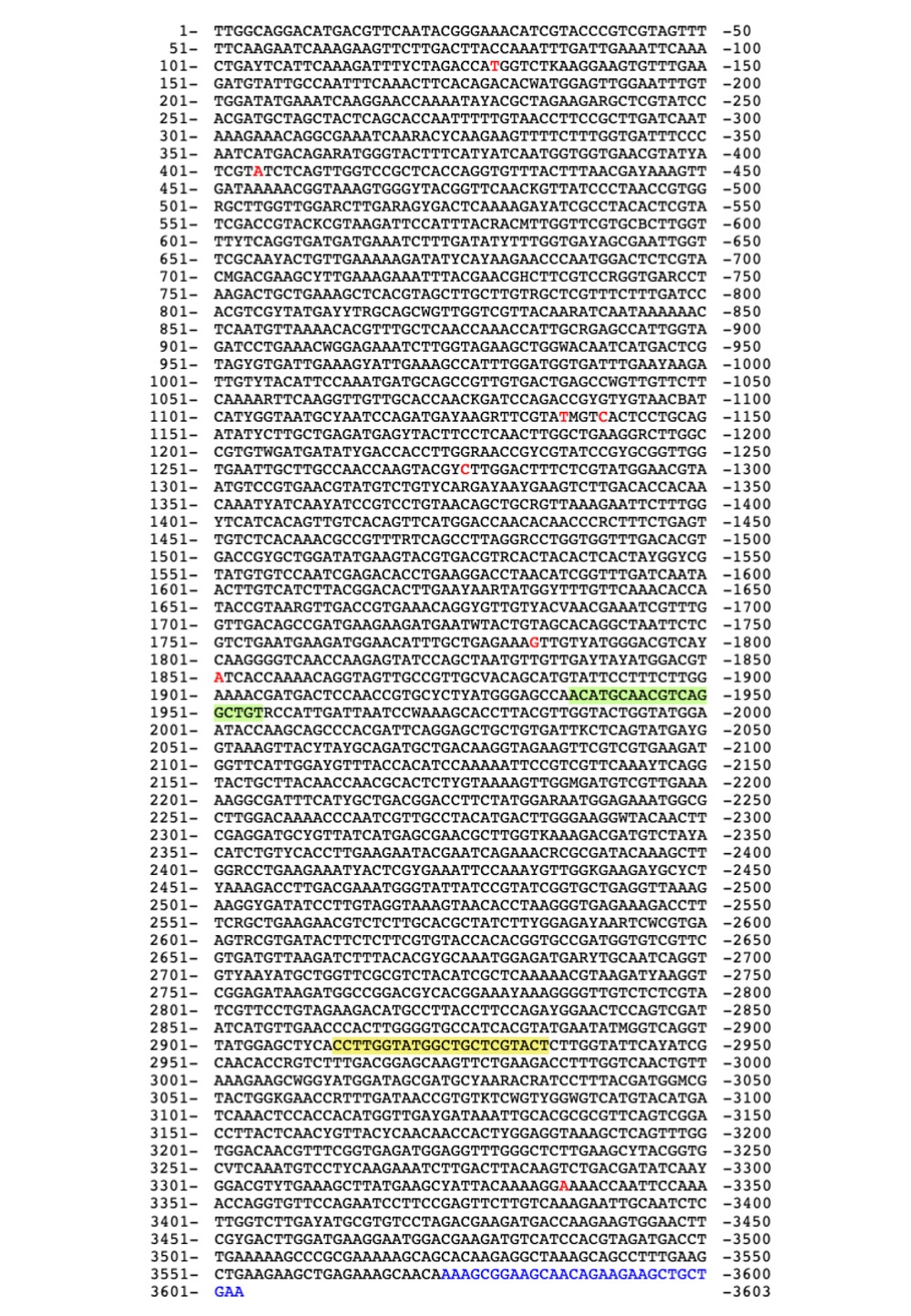

Supplement: Figure S1 — The base color code is as follows: red letters indicate that at that positions the two sequences presented differing bases, blue letters indicate positions where there are bases on the positive consensus but gaps in the negative and bold letters flank regions where there were gaps on the positive consensus but bases on the negative consensus. A degenerate base is marked as differing only if that base does not contain the corresponding base of the negative consensus. [file peerj-09-11120-s002.jpg]

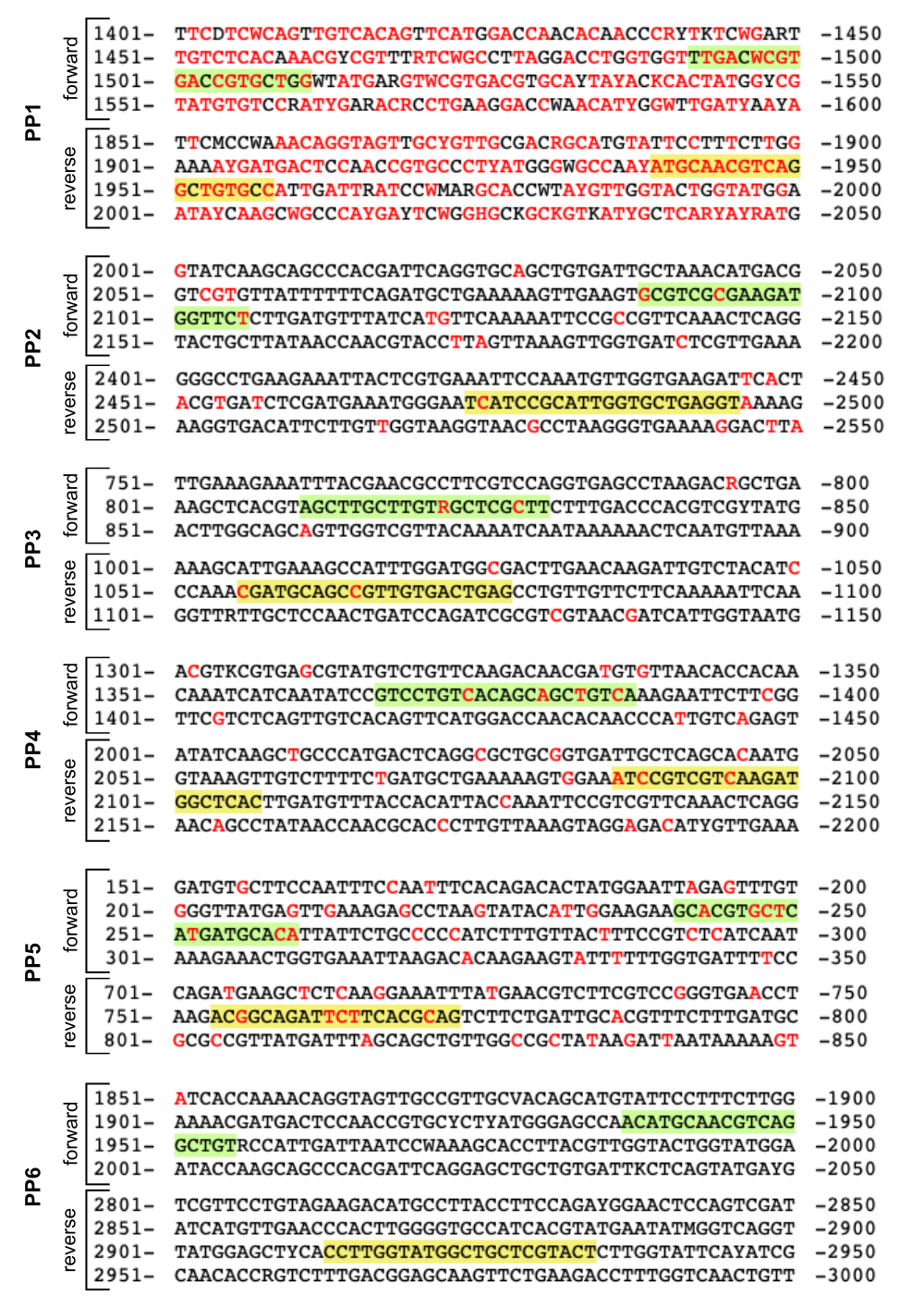

Supplement: Figure S2 — The base color code is as follows: red letters indicate that at that positions the two sequences presented differing bases, blue letters indicate positions where there are bases on the positive consensus but gaps in the negative and bold letters flank regions where there were gaps on the positive consensus but bases on the negative consensus. A degenerate base is marked as differing only if that base does not contain the correspondent base of the negative consensus. [file peerj-09-11120-s003.png]
