# Supplementary material for: PhyloPrimer: a taxon-specific oligonucleotide design platform
Source: PeerJ. 2021 Apr 29;9:e11120. doi: 10.7717/peerj.11120 (PMC8098674; doi:10.7717/peerj.11120)
Supplement: Table S1 — The reported genomes and plasmids constituted the BLAST database used to check the amplicon sequences obtained from the non-specific band observed in community A when amplified with the primer pair PP6 (Figure 5a). [file peerj-09-11120-s004.docx]

| **Species** | **GenBank genome accession numbers** |
| --- | --- |
| *Acinetobacter baumannii* | CP015121.1, CP015122.1 |
| *Enterococcus faecalis* | KB944666.1, KB944667.1, KB944668.1 |
| *Escherichia coli* | U00096.3 |
| *Klebsiella pneumoniae* | CP003200.1, CP003223.1, CP003224.1, CP003225.1, CP003226.1, CP003227.1, CP003228.1 |
| *Neisseria meningitidis* | CP021520.1 |
| *Pseudomonas aeruginosa* | AE004091.2 |
| *Staphylococcus aureus* | CP000253.1 |
| *Streptococcus agalactiae* | LR134512.1 |
| *Streptococcus pneumoniae* | UYIP01000001.1, UYIP01000002.1 |
| *Streptococcus pyogenes* | CP010450.1 |
